# Prediction of the Quantitative Biodistribution of Inhaled Titanium Dioxide Nanoparticles Using the Physiologically Based Toxicokinetic Modelling Method

**DOI:** 10.3390/toxics13100858

**Published:** 2025-10-11

**Authors:** Jintao Wang, Zhangyu Liu, Bin Wan, Xinguang Cui

**Affiliations:** 1 School of Aerospace Engineering, Huazhong University of Science and Technology, Wuhan 430074, China; 2 Research Center for Eco-Environmental Sciences, Chinese Academy of Sciences, Shuangqing RD 18, Beijing 100085, China; 3 University of Chinese Academy of Sciences, Yuquan RD 19 a, Beijing 100049, China; 4 China Astronaut Research and Training Center, State Key Laboratory of Space Medicine, Beijing 100094, China

**Keywords:** PBTK model, rat inhalation exposure, TiO_2_-NPs, hazardous nanoparticles

## Abstract

The present study aimed to establish a physiologically based toxicokinetic (PBTK) model to investigate the absorption, retention, and transport of inhaled nano-sized titanium dioxide (TiO_2_-NPs) particles in rats, thereby providing a basis for understanding the absorption, distribution, and elimination mechanisms of TiO_2_-NPs in various organs. A detailed respiratory module and the Hill coefficient equation were adopted in the PBTK model. Calibration and validation of the model were conducted using the only two available inhalation biodistribution datasets for TiO_2_-NPs found in the literature, encompassing different doses and exposure conditions. The overall fit with both datasets was acceptable with R^2^ value of 0.95 in respiratory system and 0.88 in the secondary organs. The sensitivity analysis indicated that the alveolar–interstitial transfer rate (K_alv_inter_) and tissue–blood distribution coefficients (P_lu_, P_li_, P_ki_) significantly influenced the retention of TiO_2_-NPs in pulmonary regions and distribution to secondary organs, with these parameters exhibiting time-dependent behavior. The PBTK model demonstrates a good predictive performance for TiO_2_-NPs content in all rat organs, with simulated values consistently ranging within 0.5- to 2-fold of the measured data. In last, we developed a PBTK model that can well predict the in vivo distribution of inhaled TiO_2_-NPs and provided a novel computational tool for cross-species extrapolation of human inhalation exposure and subsequent biodistribution.

## 1. Introduction

TiO_2_-NPs are extensively utilized across diverse sectors, including cosmetics, food additives, coatings, photocatalysts, and biomedical materials, attributable to their exceptional photocatalytic properties, chemical stability, and cost-effectiveness [1,2,3]. Concomitant with the advancement of nanotechnology, the escalating production of TiO_2_-NPs and their ubiquitous presence in commercial and industrial applications have inevitably led to heightened human exposure risks [4]. Routes of exposure to TiO_2_-NPs are multifaceted, encompassing oral ingestion, dermal contact, and inhalation. Notably, inhalation is recognized as the predominant and high-risk pathway in both occupational and environmental exposure scenarios [5]. While bulk TiO_2_ is generally considered chemically inert, TiO_2_-NPs exhibit distinct biological activities that diverge from their bulk counterparts. Specifically, due to their small size and high surface area, TiO_2_-NPs are known to induce oxidative stress through reactive oxygen species (ROS) generation, activate inflammasomes, and cause DNA damage, thereby raising concerns regarding their potential toxicity [6,7,8,9]. These nano-specific mechanisms can lead to a cascade of cellular responses, including inflammatory processes. Previous studies found that TiO_2_-NPs can induce similar airway inflammatory responses during both acute and subchronic exposure, and at high doses, they lead to elevated levels of IL-6 and total protein in bronchoalveolar lavage fluid [10].

The concentration of nanoparticulate matter critically influences the magnitude of its toxicological effects [11]. Moreover, the absorption, distribution, metabolism, and excretion (ADME) processes of TiO_2_-NPs are modulated by various factors, including the route of exposure, particle size, aggregation state, and surface coating [12]. It has been demonstrated that TiO_2_-NPs can enter the body via pulmonary inhalation and gastrointestinal ingestion. Following intravenous or intraperitoneal injection, TiO_2_-NPs are observed to accumulate in multiple organs, including the liver, spleen, kidneys, lungs, lymph nodes, and brain. TiO_2_-NPs are primarily excreted via the kidneys and liver [13,14]. Despite rapid excretion of most absorbed TiO_2_-NPs, complete clearance is not always achieved, which can result in their accumulation in organs such as the liver following prolonged exposure. Therefore, the accumulation of TiO_2_-NPs may pose potential risks to multiple organs, particularly the liver.

The PBTK model for hazardous nanoparticles have emerged as crucial tools in nanotoxicology research. These models are employed to evaluate the behavior of nanomaterials within the human body and their potential health risks [15,16,17,18]. The PBTK model integrates the anatomical structure, physiological functions, and hemodynamics of various tissues and organs to simulate the ADME processes of nanoparticles [19]. This model not only predicts the impacts of different exposure routes, such as inhalation, oral intake, and dermal contact, on the fate of nanoparticles in the body but also uncovers their accumulation in organs. Consequently, it provides a theoretical basis for the toxicity assessment of nanoparticles. However, accurately modeling the disposition of poorly soluble and persistent nanoparticles, such as TiO_2_-NPs, presents unique challenges for PBTK models. Key difficulties arise from their slow lung clearance kinetics, often involving phagocytic uptake by alveolar macrophages, and the potential for long-term sequestration and aggregation within tissues, which deviate from typical soluble compound kinetics. These complex interactions necessitate a more refined approach to PBTK modeling to fully capture the long-term health implications, especially following inhalation exposure. For example, Patel et al. applied the PBTK model to analyze silver nanoparticles, and their study found that the toxicity of nanoparticles is closely related to their physicochemical properties, particle size, surface modifications, and exposure routes [20]. Additionally, Ruark used the PBTK model to explore the transport and accumulation of various types of nanoparticles in the human body, pointing out that the PBTK model can effectively simulate the organ distribution characteristics of these particles and provide essential data for their long-term health risk assessment [21]. Although there are studies on the biodynamic distribution behavior of TiO_2_-NPs [22], and PBTK models have been developed for various other nanomaterials such as silver nanoparticles, gold nanoparticles, and quantum dots [23,24,25], a sufficiently established and mechanistically robust PBTK model capable of accurately predicting the complex kinetic distribution of TiO_2_-NPs in the body, particularly after inhalation exposure and incorporating key cellular processes like endo-cytosis, does not yet exist.

Given that TiO_2_-NPs have the potential to accumulate in various organs of the human body and cause toxic damage, while biodistribution studies provide valuable observational data, there remains a critical scientific gap in the development of quantitative, mechanistic models that can accurately predict their time- and dose-dependent transport and accumulation kinetics following inhalation exposure. This gap specifically pertains to robust tools capable of extrapolating across exposure scenarios and informing comprehensive risk assessment. Therefore, the objective of this study is to develop a PBTK model for TiO_2_-NPs that incorporates the complete physiological structure of the lungs. This model is designed to predict the biokinetics of TiO_2_-NPs in different organs subsequent to inhalation. Rats were selected as the research subjects for establishing the TiO_2_-NPs PBTK model. In this process, physiological and biochemical data from diverse sources were integrated. Based on rational assumptions, a series of model calibrations and optimizations were carried out. The PBTK model was calibrated and externally validated by utilizing the two available inhalation biodistribution datasets for TiO_2_-NPs. As a result, acceptable fits were achieved. Through sensitivity analysis, the alveolar—interstitial transfer rate and tissue-to-blood distribution coefficients were identified as the key determinants of lung retention and systemic distribution of TiO_2_-NPs. Our work directly addresses this by establishing a PBTK model for inhaled TiO_2_-NPs in rats, providing a quantitative framework to simulate their systemic distribution and accumulation, thereby laying the groundwork for a more predictive understanding of their long-term health implications.

## 2. Materials and Methods

### 2.1. Experimental Dataset

The model calibration dataset employed in this study was from Kreyling et al. [26]. In that work, Kreyling et al. exposed 20 adult Wistar Kyoto rats to 20 nm TiO_2_-NPs radiolabeled with 48 V via intratracheal inhalation exposure for 2 h. At 4 h, 24 h, 168 h, and 674 h post-exposure, Kreyling et al. sacrificed each animal, collected their organs and tissues, and then indirectly estimated the average mass of TiO_2_-NPs within these collected samples [26]. The specific details of the measured data used can be found in the work by Kreyling et al. [26]. The deposition fractions of TiO_2_-NPs in the rat upper respiratory tract, tracheobronchial region, and alveolar region were used as initial conditions, with these values being derived using the MPPD software (v 2.11) [27]. The initial deposition ratios of TiO_2_-NPs were extracted from graphical data using the WebPlot Digitizer (v 4.7) tool and converted into mass values [28]. The extraction was performed by two experienced operators. Based on the image resolution and refined digitalization, the extraction errors for the three deposition regions were 0.07, 0.004, and 0.0015, respectively.

### 2.2. TiO_2_-NPs PBTK Model Structure

**Figure 1:**
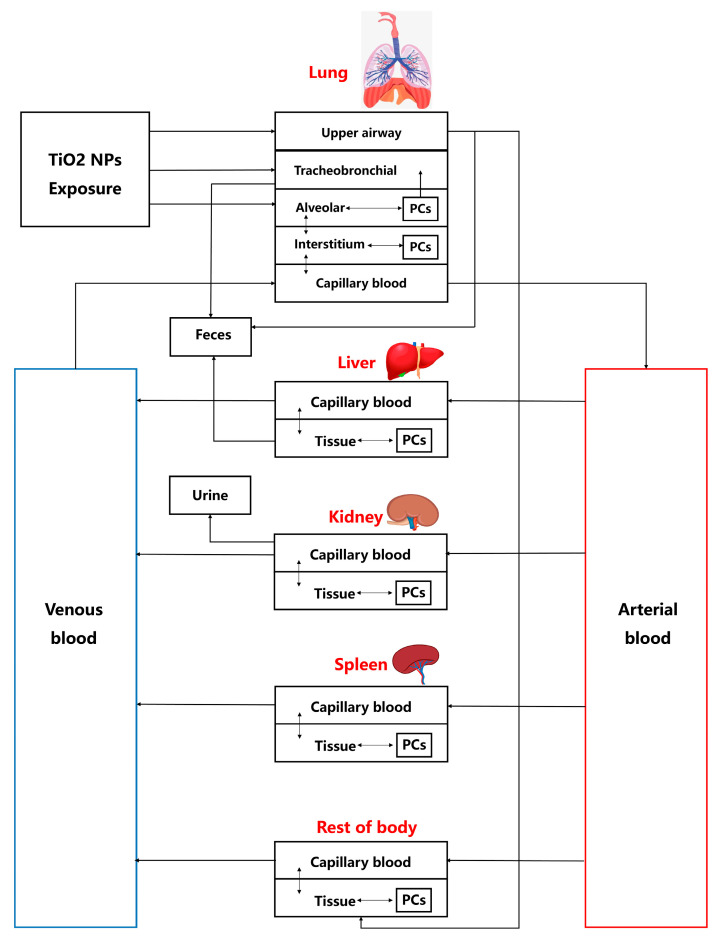
The TiO_2_-NPs PBTK model structure for rat inhalation of TiO_2_-NPs. Deposition and flow in the upper airway, tracheobronchial, and alveolar regions are related to Equations (5)–(8). Flows directed to ‘Feces’ and ‘Urine’ both represent excretory clearance processes.

Based on the absorption and distribution characteristics of TiO_2_-NPs, the organ compartments related to key kinetic processes, along with organs for which datasets are available, were selected for integration into the TiO_2_-NPs PBTK model [29,30]. The TiO_2_-NPs PBTK model includes compartments representing the lung, kidneys, liver, spleen, venous blood, arterial blood, and rest of body. Previous research indicates that permeability restricts the transfer of nanoparticles between blood and tissues [31], and that a permeability—limited model can accurately describe the biodistribution of smaller nanoparticles [31]. Consistent with these findings, the PBTK model developed in this study incorporates a permeability-limited framework, in which each compartment is divided into three sub-compartments: capillary blood, tissue, and a phagocytosis region mediated by tissue macrophages, as shown in Figure 1. The transfer of TiO_2_-NPs takes place between the capillary and tissue sub-compartments. Additionally, the transmembrane transport of TiO_2_-NPs mediates the exchange between the tissue sub-compartment and the phagocytic region. It should be noted that nanoparticles exist as thermodynamically unstable colloidal dispersions rather than solutions. By identifying saturable endocytosis of nanoparticles by tissue phagocytic cells as a critical determinant of nanoparticle tissue distribution, previous gold nanoparticle PBPK models employed the Hill equation to characterize phagocytic uptake, thereby yielding more accurate predictions [32]. In light of the absence of the Hill equation in previous TiO_2_-NPs PBTK models, this study incorporates this approach to enhance the predictive accuracy of TiO_2_-NPs biodynamics [33].

The rats are exposed to the TiO_2_-NPs oronasally, a substantial number of nanoparticles is deposited in the pulmonary region, with only a small fraction crossing the blood–air barrier, which consists of capillary endothelial cells and alveolar epithelial cells, to enter the circulatory system [33,34]. In the TiO_2_-NPs PBTK model, the lungs are divided into four compartments: the tracheobronchial region, alveolar region, lung region, and pulmonary capillary region. In the tracheobronchial compartment, nanoparticles remain in a “free” state, whereas in the alveolar and lung, they can be engulfed by macrophages. The TiO_2_-NPs enter the rat system through three primary compartments in the lungs: the tracheobronchial region, the alveolar region, and the upper respiratory tract region. The excretion of TiO_2_-NPs occurs through three pathways: (a) in the liver, where nanoparticles are excreted via the hepatic–biliary system into the feces, (b) in the kidneys, where nanoparticles are excreted into the urine, and (c) through tracheobronchial clearance [15,35]. More details can be seen in Figure 1, which showed the TiO_2_-NPs PBTK model structure.

### 2.3. Mathematical Description of the TiO_2_-NPs PBTK Model

**Figure 2:**
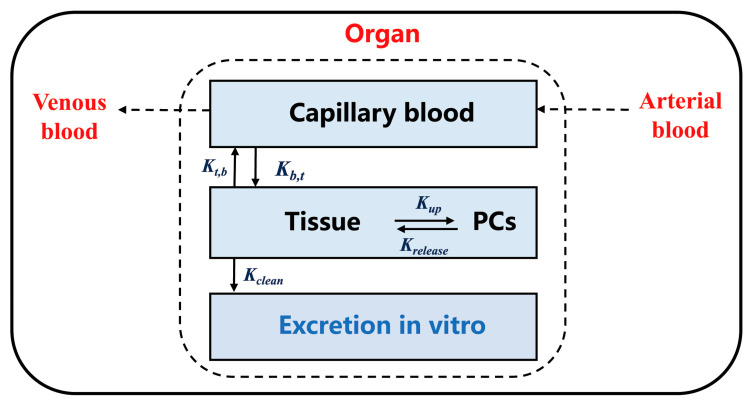
Schematic diagram of the sub-compartmental structure of each organ in the TiO_2_-NPs PBTK model. Parameters “$K_{t,b}$” and “$K_{b,t}$” represent the flow between capillary blood and tissue, while parameters “$K_{\mathrm{up}}$” and “$K_{\mathrm{release}}$” represent the flow between tissue and PCs. These flows are applicable to Equations (1)–(3).

As shown in Figure 2, the interior of each organ contains capillary space and tissue space, and the transfer of TiO_2_-NPs between any two sub-compartments can be expressed as:

(1) $$\frac{dM_{o\;,\mathrm{cab}}}{\mathrm{dt}}={\;Q}_{o}\times \left(\frac{M_{\mathrm{art}}}{V_{\mathrm{art}}}-\frac{M_{o\;,\mathrm{cab}}}{V_{o,\;\mathrm{cab}}}\right)-{\;X}_{o}\times Q_{o}\times \left(\frac{M_{o\;,\mathrm{cab}}}{V_{o,\;\mathrm{cab}}}-\frac{M_{o\;,\mathrm{tis}}}{P_{o}\cdot V_{o,\;\mathrm{tis}}}\right)$$

(2) $$\frac{dM_{o\;,\mathrm{tis}}}{\mathrm{dt}}=X_{o}\times Q_{o}\times \left(\frac{M_{o\;,\mathrm{cab}}}{V_{o,\;\mathrm{cab}}}-\frac{M_{o\;,\mathrm{tis}}}{P_{o}\cdot V_{o,\;\mathrm{tis}}}\right)-\;(K_{o,\;\mathrm{up}}\times {\;M}_{o\;,\mathrm{tis}}-{\;K}_{o,\;\mathrm{out}}\times \;M_{o,\;\mathrm{PCs}})$$

(3) $$\frac{dM_{o\;,\mathrm{PCs}}}{\mathrm{dt}}=K_{o,\;\mathrm{up}}\times {\;M}_{o,\;\mathrm{tis}}-{\;K}_{o,\;\mathrm{out}}\times \;M_{o,\;\mathrm{PCs}}$$

where $M_{o,\;\mathrm{cab}}$ (ng), $M_{o,\;\mathrm{tis}}$ (ng) and $M_{o,\;\mathrm{PCs}}$ (ng) are the mass of TiO_2_-NPs in the capillary blood of the organ, the tissue of the organ and the phagocytic cells (PCs) of the organ, respectively. $M_{\mathrm{art}}$ (ng) and $V_{\mathrm{art}}$ (L) are is the mass and volume of TiO_2_-NPs in the arterial blood. The physiological parameters, $Q_{o}$ (L/h) is the blood flow to the organ; $Q_{c}$ (L/h) is the cardiac output; $V_{\mathrm{art}}$ (L), $V_{o,\;\mathrm{cab}}$ (L), and $V_{o,\mathrm{tis}}$ (L) are the volume of the arterial blood, the capillary blood of the organ and the tissue of the organ, respectively, and can be obtained from the literature based on the weight of the rat [36]. $X_{o}$ (unitless) is the permeability coefficient between capillary blood and tissue, and $P_{o}$ (unitless) is the ratio of tissue and blood distribution coefficients for the organ. $K_{o,\;\mathrm{out}}$ (per h) is the release rate constant of TiO_2_-NPs from PCs to the tissue in the organ, and $K_{o,\;\mathrm{up}}$ (per h) is the uptake rate of TiO_2_-NPs from the tissue to PCs in the organ. TiO_2_-NPs are assumed to be taken by PCs via endocytosis, which described by the uptake rate as [37,38]:

(4) $$K_{o,\;\mathrm{up}}\;=\;\frac{K_{o,\;\max\;}\times \;t^{n_{0}}}{K_{o,\;50}^{n_{0}}\;+{\;t}^{n_{0}}}$$

where t (h) is the simulation time, $K_{o,\;\max}$ (per h) is the maximum uptake rate parameter in the organ, $K_{o,\;50}^{n_{0}}$ (h) is the time reaching half of $K_{o,\;\max\;}$, and $n_{0}$ (unitless) is the hill coefficient. The *o* in the parameter subscript represents lung (lu), spleen (spl), liver (li), kidney (ki), and rest of body (rob). By solving the above equations, the mass of TiO_2_-NPs in major organs can be obtained.

Nanoparticles deposited in the lungs, due to gravitational settling, will primarily deposit in the upper respiratory tract, tracheobronchial region, and alveolar region. Given that the nature of inhalation exposure dictates that most TiO_2_-NPs will be deposited in the pulmonary regions, in the TiO_2_-NPs PBTK model, TiO_2_-NPs exposed via inhalation will be deposited in the upper respiratory tract, tracheobronchial region, and alveolar region. Consequently, the equations for the different lung compartments are as follows:

(5) $$\frac{dM_{\mathrm{upp}}}{\mathrm{dt}}=\;EC\times BR\times {\;\mathrm{FR}}_{\mathrm{upp}}\;-{\;K}_{\mathrm{upp}\text{-}\;\mathrm{rob},\;\mathrm{tis}}\times M_{\mathrm{upp}}\;-\;K_{\mathrm{upp}\text{-}\mathrm{feces}}\times M_{\mathrm{upp}}$$

(6) $$\frac{dM_{\mathrm{tra}}}{\mathrm{dt}}=EC\times BR\times {\;\mathrm{FR}}_{\mathrm{tra}}\;-\;K_{\mathrm{alv},\;\mathrm{PCs}\text{-}\mathrm{tra}}\times M_{\mathrm{alv},\;\mathrm{PCs}}-K_{\mathrm{tra}\text{-}\;\mathrm{feces}}\times M_{\mathrm{tra}}$$

(7) $$\frac{dM_{\mathrm{alv},\mathrm{PCs}}}{\mathrm{dt}}={\;K}_{\mathrm{alv},\;\mathrm{up}}\times {\;M}_{\mathrm{alv}}\;-\;{\;K}_{\mathrm{alv},\;\mathrm{out}}\times M_{\mathrm{alv},\;\mathrm{PCs}}-K_{\mathrm{alv},\;\mathrm{PCs}\text{-}\;\mathrm{tra}}\times M_{\mathrm{alv},\;\mathrm{PCs}}$$

(8) $$\frac{dM_{\mathrm{alv}}}{\mathrm{dt}}=EC\times BR\times {\;\mathrm{FR}}_{\mathrm{alv}}+{(K}_{\mathrm{lung},\;\mathrm{tis}\text{-}\mathrm{alv}}\times M_{\mathrm{lung},\;\mathrm{tis}}-K_{\mathrm{alv}\text{-}\mathrm{lung},\;\mathrm{tis}}\times M_{\mathrm{alv}})-{(K}_{\mathrm{alv},\;\mathrm{up}}\times M_{\mathrm{alv}}-K_{\mathrm{alv},\;\mathrm{out}}\times M_{\mathrm{alv},\;\mathrm{PCs}})$$

where EC (ng/L) represents the exposed TiO_2_-NPs aerosol mass concentration, BR (L/min) represents breathing volume intake rate of the rats.${\;\mathrm{FR}}_{\mathrm{upp}}$, ${\;\mathrm{FR}}_{\mathrm{tra}}$, and ${\;\mathrm{FR}}_{\mathrm{alv}}$ represent the proportion of the initial inhalation dose of TiO_2_-NPs deposited in the upper respiratory tract, tracheobronchial region, and alveolar region, respectively. $K_{\mathrm{upp}\text{-}\;\mathrm{rob},\;\mathrm{tis}}$ (per h) denotes the transfer rate of TiO_2_-NPs from the upper respiratory tract to the ROB tissue; $K_{\mathrm{upp}\text{-}\;\mathrm{feces}}$ (per h) denotes the transfer rate of TiO_2_-NPs from the upper respiratory tract to the feces; $K_{\mathrm{alv},\;\mathrm{PCs}\text{-}\mathrm{tra}}$ (per h) represents the transfer rate of TiO_2_-NPs from the alveolar phagocytic region to the tracheobronchial region; $K_{\mathrm{tra}\text{-}\;\mathrm{feces}}$ (per h) represents the transfer rate of TiO_2_-NPs from the tracheobronchial region to the feces; ${\;K}_{\mathrm{alv},\;\mathrm{up}}$ (per h) and ${\;K}_{\mathrm{alv},\;\mathrm{out}}$ (per h) represent the transfer rates between the alveolar and alveolar phagocytic regions, respectively; $K_{\mathrm{lung},\;\mathrm{tis}\text{-}\mathrm{alv}}$ (per h) and $K_{\mathrm{alv}\text{-}\mathrm{lung},\;\mathrm{tis}}$ (per h) represent the transfer rates between the alveolar region and lung tissue, respectively. All equations were solved in Matlab SimBiology [37].

### 2.4. Model Parameterization

In the PBTK modeling of TiO_2_-NPs, the parameters are categorized into two types: physiological parameters and chemistry-specific parameters [39,40,41]. Physiological parameters are derived through experimental measurements, retrieved from published literature, or calculated based on original published research [40]. These parameters include average body weight (BW), total cardiac output (Qcc), tissue volume fractions and blood flow fractions for organs such as the liver, kidneys, spleen, and other body tissues (Table 1). The chemical-specific parameters for rats, in conjunction with their corresponding physiological parameters, are initialized either based on previous PBTK models or fitted experimental datasets (Table 2). It is assumed that the physiological parameters remain constant over time. For the adjustment of chemistry-specific parameters, further fitting was performed using the Matlab App Simbiology (R2024a) [37]. The slider tool was applied to adjust these parameters, ensuring that the model results are in accordance with the experimental data for TiO_2_-NPs.

### 2.5. The TiO_2_-NPs PBTK Model Validation

Another content–time dataset obtained from a single 6 h inhalation exposure to 20 nm TiO_2_-NPs was employed as the validation dataset [42]. The model was validated by comparing its output with the actual experimental results. Considering that the calibration and validation datasets were collected under different experimental conditions, the PBPK modeling guidelines from the World Health Organization [43] indicate that a model is considered valid and reasonable when the simulated outcomes align with the experimental kinetic curves and generally fall within a range of 0.5 to 2 times the experimental data. The performance of TiO_2_-NPs PBTK model was assessed using the Absolute Average Fold Error (AAFE), calculated as follows:

(9) $$\mathrm{AAFE}\;={\;10}^{\frac{1}{n}\sum |\log(\frac{\mathrm{simulated}}{\mathrm{measured}})|}$$

where *n* represents the total number of data points. It is generally accepted when AAFE ≤ 2, where the predicted values exceed the measured values [37].

### 2.6. Sensitivity Analysis

To identify the parameters that most significantly influence the distribution of TiO_2_-NPs in each compartment, a sensitivity analysis was conducted on the chemistry-specific parameters in the tissue and phagocytic sub-compartments of each organ within the model [44]. The sensitivity coefficient is determined using the following equation:

(10) $$S_{\mathrm{ij}}^{\mathrm{rel}}\left(t\right)=\frac{\partial y_{i}(t)}{\partial \theta_{i}}\cdot \frac{\theta_{i}}{y_{i}(t)}=\frac{\partial \ln(y_{i}(t))}{\partial \ln(\theta_{i})}$$

where $y_{i}(t)$ is the target output variable and $\theta_{i}$ is the i-th parameter in the model. The sensitivity parameters are calculated using the “sbioselect” function.

## 3. Results

### 3.1. TiO_2_-NPs PBTK Model Prediction

**Figure 3:**
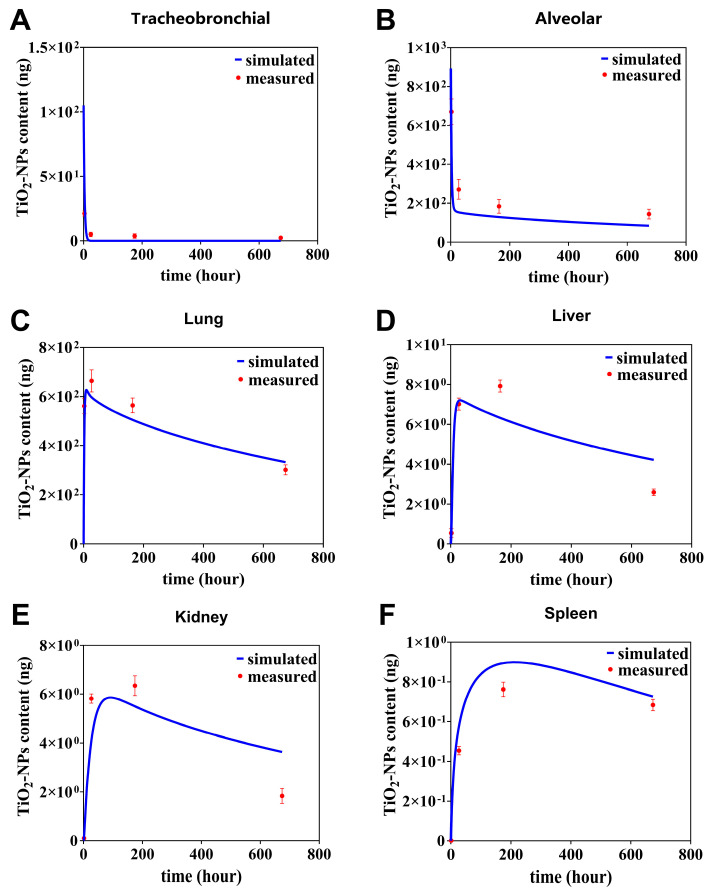
TiO_2_-NPs content in tracheobronchial (**A**), alveolar (**B**), lung (**C**), liver (**D**), kidney (**E**), spleen (**F**) over time, with simulated and measured data. The measured data (red dots) denote mean values ± standard deviation (SD).

The TiO_2_-NPs PBTK model was used to gain the absorption and distribution of TiO_2_-NPs in rats following inhalation exposure. As shown in Figure 3, the trends of simulated TiO_2_-NPs content in different organs over time (blue curve) are fit with the actual measured data (red dots), which confirms the capability of the PBTK model to predict the variation of TiO_2_-NPs content across various regions.

From Figure 3, we further find that a rapid absorption followed by a gradual dissipation of the predicted curve TiO_2_-NPs in the primary organs such as tracheobronchial, and alveolar (Figure 3A,B). For instance, in the tracheobronchial compartment, the content sharply declined from 40 ng at 0 h to 0.12 ng at 21 h. Similarly, in the alveolar region, it decreased from 892 ng at 0 h to 158 ng at 28 h. Afterwards, the simulated curve kept relatively stable from 28 h to 674 h in tracheobronchial compartment. In alveolar compartment, it decayed slowly from 28 to 674 h, with the slow decay rate being 17% of the fast decay rate. The deviation between simulated and observed values was quantitatively assessed using the relative error (RE), which is calculated as follows: $RE\left(\%\right)=\frac{\left|Simulated\;value\;-Measured\;value\right|}{easured\;value}\times 100\%.$ This metric was used to evaluate the agreement at each specific data point. It was observed that the simulated data at 4, 24, 168, and 674 h deviated from the measured data by 15%, 11%, 9.8%, and 5% in the tracheobronchial, and by 28%, 33%, 29%, and 25% in the alveolar, respectively (Figure 3A,B). In contrary, in lung compartment, it can be predicted the simulated curve quickly rises the peak value of 625 ng at 10 h, then decreases half of the peak at 721 h (Figure 3C). It was observed that the simulated data at 4, 24, 168, and 674 h deviated from the measured data by 24%, 9%, 10% and 10%.

In the secondary organs, the simulated content–time curves of TiO_2_-NPs in the liver and kidney compartments exhibited similar trends (Figure 3D,E). In liver compartment, the simulation curve predicts a peak of 7.2 ng at 28 h and then a slow decay to half of the peak at 871 h. In kidney compartment, the simulation curve predicts a peak of 5.86 ng at 90 h and then a slow decay to half of the peak at 957 h. It was observed that the simulated data at 4, 24, 168, and 674 h deviated from the measured data by 33%, 2.5%, 16% and 38% in the liver, and by 33%, 29%, 13% and 43% in the kidney, respectively. In contrast to this, in spleen compartment, the simulation curve predicts a peak of 0.89 ng at 17 h and then a slow decay to 19% of the peak at 1454 h. The simulated values of the simulation curves at 4, 24, 168 and 674 h differed from the measured values by 17%, 21%, 14% and 6%, respectively (Figure 3F).

**Figure 4:**
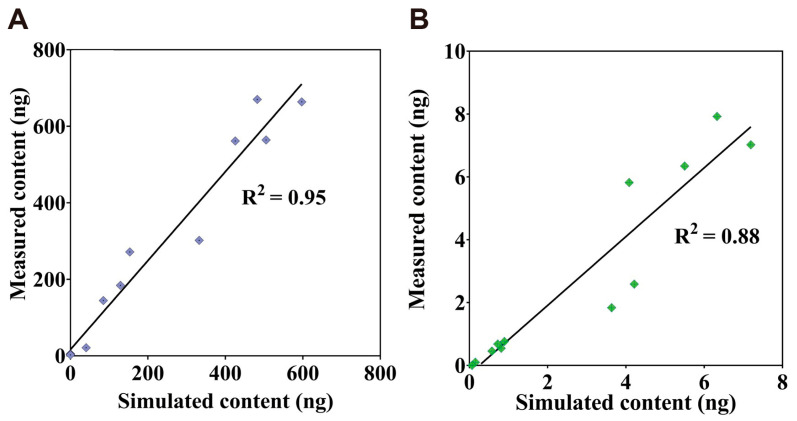
Correlation between simulated and measured TiO_2_-NPs content. (**A**) present the correlation analysis among alveoli, lung, and tracheobronchial. (**B**) presents the correlation analysis among the liver, spleen, and kidney.

To further evaluate the accuracy of the PBTK model, it illustrates the correlation between the predicted content of TiO_2_-NPs obtained using the TiO_2_-NPs PBTK model and the experimentally measured values in Figure 4. In Figure 4A, a strong correlation is observed between the simulated and measured content in the alveoli, tracheobronchial, and lung, with an R^2^ value of 0.95. As shown in Figure 4B, the correlation for the liver, kidney, and spleen was slightly lower, with an R^2^ value of 0.88. Nevertheless, since the R^2^ values for all lung regions and secondary organs exceeded 0.85, the model demonstrates good overall predictive performance.

In summary, the present PBTK model can effectively capture the distribution patterns and temporal dynamics of TiO_2_-NPs across various organs, with a particularly good predicted effect in different regions of the lungs.

### 3.2. The TiO_2_-NPs PBTK Model Evaluation with an Independent Data

**Figure 5:**
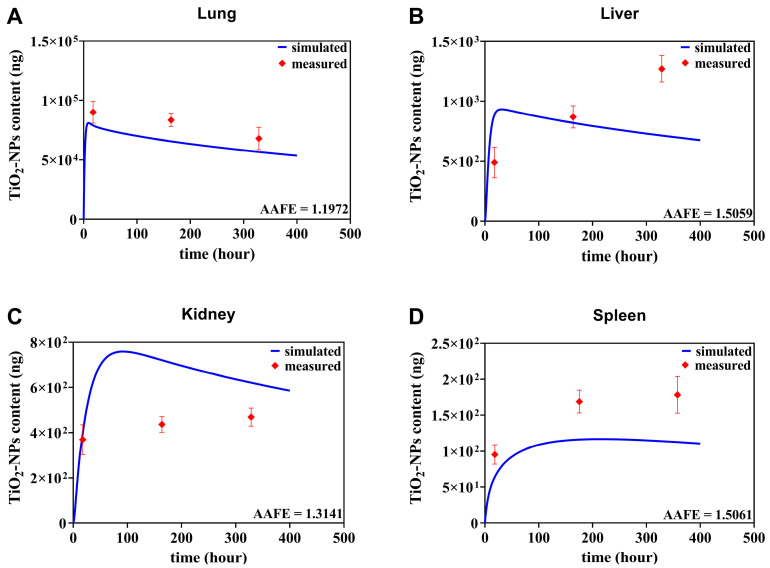
Comparative assessment of TiO_2_-NPs PBTK model predictions and experimental biological distribution data from Gosens et al. [42]. Quantitative comparison of TiO_2_-NPs PBTK simulation results with measured content of TiO_2_-NPs in lung, liver, kidney, and spleen following single nasal exposure at a mass content of 20 mg/m^3^ in rat models.

In this section, an independent dataset was used to further validate the TiO_2_-NPs PBTK model [42]. This dataset was obtained from a single 6 h inhalation exposure to Wistar rat 20 nm TiO_2_-NPs was employed as the validation dataset [42], which is described in detail in Section 2.5. It is important to note that no calibration parameters of the PBTK model were re-adjusted or re-fitted during these independent validation steps. As shown in Figure 5A, the predicted content–time curve for the lungs aligns well with the experimental data, both exhibiting a rapid increase from 0 to 24 h followed by a gradual decline afterwards. In the secondary organs, differences in inhalation exposure conditions and dosages led to a continuous increase in measured TiO_2_-NPs content in the liver, resulting in a distinct trend (Figure 5B). However, the simulated data remained in reasonable proximity to the measured data. Moreover, the predicted trends and magnitudes for both the kidney (Figure 5C) and spleen (Figure 5D) closely matched the measured data. Despite variations in exposure methods, dosages, and nanoparticle types that may influence nanoparticle biodistribution, the model’s predictive performance is considered acceptable, given that the AAFE (defined in Section 2.5) for the lung, liver, kidney, and spleen were 1.1972, 1.5059, 1.3141, and 1.5061, respectively, all falling well below the commonly accepted threshold of 2 for PBTK models.

### 3.3. The TiO_2_-NPs PBTK Model Sensitivity Analysis

**Figure 6:**
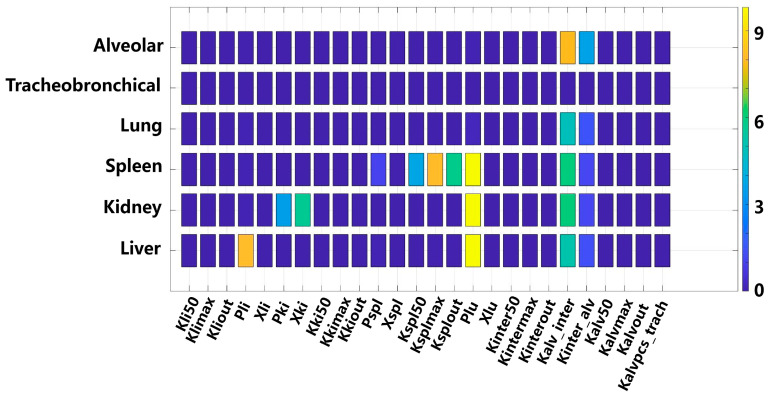
Parameter sensitivity analysis for organ compartments in the TiO_2_-NPs PBTK model.

The sensitivity analysis of the PBTK model parameters is depicted in Figure 6. For the alveolar and lung compartments, the transfer coefficient between the alveolar and lung regions (K_alv_inter_, sensitivity coefficient = 7.8) emerged as the most sensitive parameter, which is as high as the distribution coefficient between pulmonary tissue and blood (P_lu_). This finding implies that TiO_2_-NPs deposited in the alveolar region are prone to translocating across the alveolar epithelium into the lung, thereby causing a rapid increase in the TiO_2_-NPs content within this compartment. In contrast, there is no significant sensitive to all parameters for the tracheobronchial region, suggesting that the transfer of TiO_2_-NPs from the alveolar phagocytic region to the tracheobronchial region is minimal. Conversely, for the tracheobronchial region, no significant sensitivity was observed for all parameters. This suggests that the transfer of TiO_2_-NPs from the alveolar phagocytic region to the tracheobronchial region is negligible. Regarding the liver, kidneys, and spleen, the parameter P_lu_ (sensitivity coefficient = 9.5) was identified as the most sensitive parameter, underscoring its crucial role in the systemic distribution of TiO_2_-NPs. Additionally, the liver-to-blood distribution coefficient (P_li_, sensitivity coefficient = 8.2) exhibited notable sensitivity for the hepatic compartment. For the spleen, the parameter governing interactions between the splenic tissue compartment and the phagocytic subcompartment showed the highest sensitivity.

**Figure 7:**
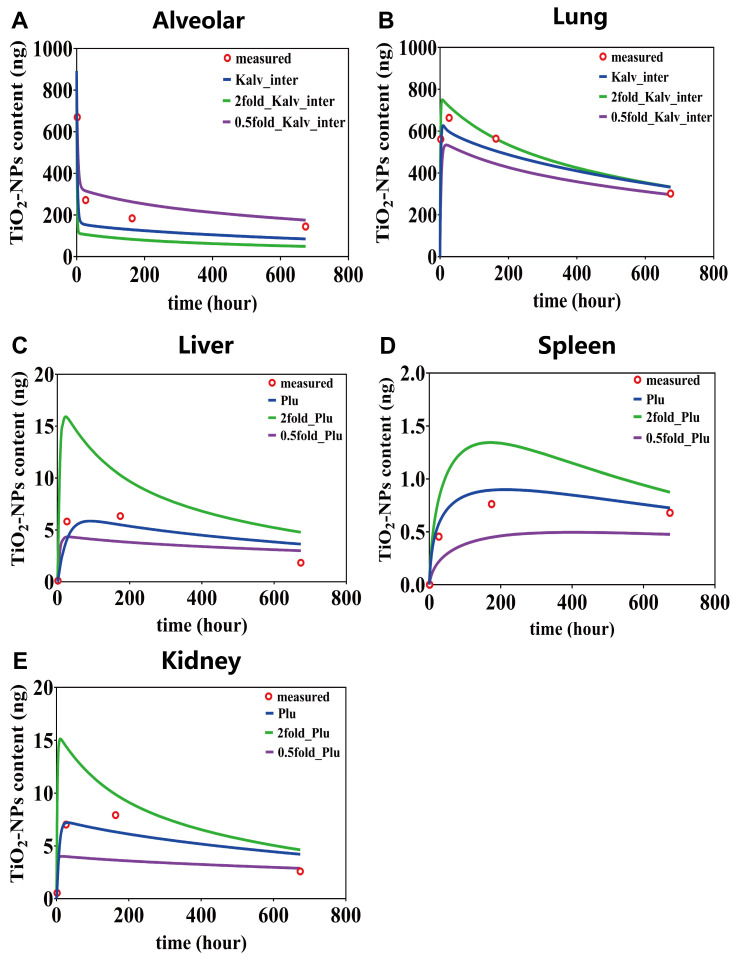
The content–time curves for alveolar (**A**), lung (**B**), liver (**C**), spleen (**D**) and kidney (**E**), based on the most influential parameters identified through sensitivity analysis.

Based on the local sensitivity analysis results presented in Figure 6, for the most sensitive parameters in each compartment, we carried out parameter perturbations through 2 fold amplification and 0.5 fold reduction. Subsequently, we observed the consequent changes in the predictive curves. It is found that the parameter K_alv_inter_ (alveolar-to-interstitial transfer rate) similarly influences the content–time curves of lung (Figure 7A,B). Conversely, its impact on alveolar content–time curves presents a distinctly different pattern: minimal effect within the first 7 days, gradually increasing with prolonged simulation time. In the alveolar region, the predictions using 2 fold-K_alv_inter_ deviated from the four experimental values by 61%, 4%, 41%, and 80%, respectively, while those using 0.5 fold-Kalv_inter showed deviations of 2%, 16%, 42%, and 21%. In the overall lung region, the deviations between the 2 fold-K_alv_inter_ predictions and the experimental values were 11%, 8%, 1%, and 11%, whereas the 0.5 fold-K_alv_inter_ predictions differed by 59%, 20%, 22%, and 2%, respectively.

The parameter P_lu_ plays a crucial role in determining the content–time curves of liver, spleen, and kidney (Figure 7C–E). The sensitivity exhibits a pronounced time-dependency, with parameter variations exerting the most substantial impact during the initial exposure days, progressively diminishing as simulation time advances. Notably, the content of TiO_2_-NPs in the spleen exhibits a more evident dose—dependent characteristic. Modifications to the parameter Plu lead to substantial differences in the predictive curves. For instance, when P_lu_ was expanded from 0.5 to 2 times, the simulation curve showed a change from steady to decreasing. In the liver compartment, predictions using the 2 fold-P_lu_ deviated from the four experimental measurements by 1372%, 99%, 23%, and 79%, respectively, while those using the 0.5 fold-Plu differed by 293%, 43%, 54%, and 11%. In the spleen compartment, the deviations for the 2 fold-P_lu_ were 0%, 72%, 76%, and 28%, whereas the 0.5 fold-P_lu_ resulted in deviations of 0%, 56%, 45%, and 31%. In the kidneys, the predicted values with the 2 fold-P_lu_ deviated by 2220%, 172%, 63%, and 160%, while the 0.5 fold-P_lu_ showed deviations of 437%, 25%, 40%, and 63%.

In summary, the time-dependent parameter sensitivity primarily reflects the dominant kinetic processes across different exposure stages. For example, the Plu greatly influences secondary organ accumulation during early exposure. When Plu was doubled, the deviation in liver content reached 1372% on day 1, but decreased to less than 100% by day 7, indicating that its impact diminishes as equilibrium is approached. In contrast, the Kalv_inter shows minimal effect within the first seven days, with deviations in alveolar content of no more than 4%, but its influence increases noticeably at later time points, highlighting the complex and delayed dynamics of nanoparticle transport and clearance.

## 4. Discussion

In this study, we developed a predictive PBTK model for TiO_2_-NPs, integrating tissue-specific endocytosis mechanism characterized by the Hill coefficient [32]. The model was meticulously calibrated and simulated using detailed time-resolved organ distribution data obtained from rats following a single inhalation exposure to 20 nm TiO_2_-NPs [26]. Rigorous validation against experimental data from Gosens et al. demonstrated the predictive capabilities of the TiO_2_-NPs PBTK model [42]. Through systematic sensitivity analysis, we elucidated that the lung-to-blood distribution coefficient (P_lu_) and alveolar-to-lung transfer rate (K_alv_inter_) emerged as pivotal parameters significantly modulating TiO_2_-NPs distribution across secondary organs and pulmonary structures. Our findings not only validate the PBTK model’s efficacy in predicting TiO_2_-NPs biodistribution in rat models but also provide critical mechanistic insights for developing prevention strategies for inhaled hazardous nanoparticles.

Hazardous nanoparticles are recognized to have detrimental impacts on human health, and their toxic potential is closely associated with the exposure concentration [45]. To more accurately evaluate these risks, the PBTK model has been developed to predict the complex distribution of these particles within organs and tissues [30]. In occupational exposure settings, inhalation is identified as the primary route of entry, whereas systemic absorption via dermal penetration is generally considered negligible. Given the characteristics of inhalation exposure, the majority of nanomaterials are either deposited in pulmonary structures or excreted, with only a small portion successfully traversing the air–blood barrier. Consequently, an advanced pulmonary compartmental model was proposed. This model subdivides the lung architecture into four distinct sub-chambers: the tracheobronchial region, alveolar region, lung tissue, and lung capillary blood. Additionally, macrophage sub-compartments were incorporated into the alveolar and lung tissue to simulate phagocytic endocytosis mechanisms. The simulation results showed a high degree of agreement between the predicted concentrations and the experimental data, revealing consistent trends and temporal content distributions (Figure 3A–C). These findings validate the proposed compartmental structure, effectively depicting the complex dynamics of deposition and rapid clearance of inhaled nanoparticles.

Sensitivity analysis revealed that the parameter alveolar-to-interstitial transfer rate (K_alv_inter_) plays a critical role in influencing the simulated contents of TiO_2_-NPs in both the alveolar region and the lung. This parameter directly influences the residence time of nanoparticles in the alveolar and governs their translocation into lung tissue and systemic circulation. Equally important is the influence of the parameter lung-to-blood distribution coefficient (P_lu_) on TiO_2_-NP exposure levels in secondary organs such as the liver, kidney and spleen. As a key parameter in the inhalation exposure pathway, variations in P_lu_ influences the proportion of nanoparticles transitioning from the lungs into the bloodstream. The magnitude of P_lu_ reflects the affinity of lung tissue for the particles and thereby modulates systemic distribution. Given that hepatic uptake is blood-flow-dependent, renal clearance relies on filtration, and splenic removal is mediated by the mononuclear phagocyte system, even slight changes in Plu can substantially alter biodistribution patterns in these organs. Specifically, the Time-Integrated Amount in System (TIAS) for Kalv_inter (5288.4) and Plu (6441.0) directly support and quantify this regulatory capacity, as these parameters dictate the fraction and rate of nanoparticles retained within the lung versus those translocated systemically. These findings are consistent with the ‘regulatory role’ of pulmonary retention on systemic exposure reported by Chou et al. [24].

In addition, we conducted a focused examination of the in vivo behavioral differences between TiO_2_-NPs and titanium ions. A comparative analysis of published data [46] and our model calculations unveiled remarkable differences in organ distribution patterns, clearance pathways, and elimination rates, highlighting fundamentally divergent biological behaviors. Titanium ion, being small hydrophilic solutes, can easily diffuse throughout the body via the circulatory system. This pharmacokinetic characteristic accounts for their rapid renal accumulation and short systemic half-life subsequent to intravenous administration, a pattern that is in line with what has been observed for numerous other metal ions [47]. In contrast, TiO_2_-NPs, due to their particulate nature, display biological behaviors that are highly dependent on interactions with host cells, particularly components of the mononuclear phagocyte system [48]. Once entering systemic circulation, TiO_2_-NPs are preferentially sequestered by Kupffer cells in the liver and macrophages in the spleen, primarily through phagocytic uptake. This clearance mechanism is fundamentally distinct from the renal filtration pathway responsible for titanium ion elimination [46]. Upon internalization, TiO_2_-NPs have a propensity to form intracellular aggregates. These aggregates are refractory to metabolism and translocation, frequently remaining within organelles like lysosomes for extended durations. This protracted intracellular retention leads to an extended tissue half-life of TiO_2_-NPs. It also gives rise to concerns about potential long-term biological effects under chronic exposure scenarios.

A crucial consideration in the PBTK modeling of nanoparticles, beyond their transport and retention, is the distinction between the behavior of the intact particulate form and any potential dissolved ionic species. While some inorganic nanoparticles can undergo dissolution in vivo, understanding whether the observed biodistribution is primarily driven by the nanoparticle itself or by released ions is vital for accurate risk assessment and for developing models focused on the unique characteristics of nanoparticles. The mechanistic differences between TiO_2_-NPs and titanium ions were further substantiated by half-life calculations based on data from Kreyling et al. [49]. TiO_2_-NPs exhibited substantially longer half-lives in the liver, kidneys, and spleen—452.8 h, 179.6 h and 350.7 h, respectively—compared to those of titanium ions, which were only 1.9 h, 3.3 h, and 2.1 h in the same organs [46]. These conspicuous discrepancies underscore that the in vivo behavior of nanoparticles cannot be comprehensively elucidated merely by their chemical composition. Instead, it is essential to consider their physical form and the impact it exerts on cellular processes, including uptake and intracellular trafficking. Consequently, nanoparticles exhibit significant differences from their ionic counterparts with respect to cellular uptake mechanisms, clearance pathways, and retention times [46]. This finding emphasizes the need to avoid extrapolations based on metal ions when evaluating the toxicological behavior of nanomaterials. Instead, their unique particle-specific biological interactions must be taken into consideration. In particular, within the context of inhalation exposure models, our results suggest that TiO_2_-NPs enter systemic circulation predominantly in particulate form, exhibiting biodistribution patterns more akin to those observed following intravenous injection of nanoparticles, rather than those of freely dissolved ions, see Table 3.

Based on the above significant differences, it is widely recognized that PBTK model for nanoparticles exhibit fundamental distinctions from those established for small molecules [13]. In the domain of nanoparticle PBTK model, gold nanoparticles have emerged as the most extensively studied nanomaterial due to their exceptional stability and metabolic inertness within biological systems [33,37]. Lin et al. conducted a comprehensive comparative analysis of 14 distinct PBTK model structures, identifying a membrane-limited endocytosis model incorporating Hill coefficient descriptions as the most predictively robust approach [32]. Subsequent research has progressively leveraged Hill coefficient equations to characterize organ-specific endocytosis mechanisms, consistently demonstrating remarkable predictive performance [32,50]. However, a critical research gap existed wherein Hill coefficient equation had not been systematically applied to titanium dioxide PBPK modeling. The present study addresses this scientific void, successfully predicting the biomechanical behavior of inhaled titanium dioxide in rat models. The clinical implications of inhaled TiO_2_-NPs in humans remain limited by scarce case reports and epidemiological data. Occupational exposure studies indicate subtle lung inflammation, oxidative stress, and elevated DNA damage markers in TiO_2_ manufacturing workers, supporting our PBTK model’s prediction of high pulmonary accumulation in rats [51,52]. However, large cohort studies show no significant lung cancer risk, contrasting with rat inhalation studies reporting lung tumors [53]. These findings highlight the translational potential of our model for predicting human-relevant risks and underscore the need for further human studies to validate these predictions.

Despite its significant contributions, this study acknowledges inherent limitations of the PBTK model, particularly concerning its structural and parameter-related assumptions. The current model employs a simplified compartmental structure, treating organs as homogeneous entities, which may overlook the complex microenvironment and sub-cellular distribution of nanoparticles. Furthermore, parameter estimation was primarily based on single-exposure data with a limited number of time points. This limitation can affect prediction accuracy, especially for nanoparticle-specific kinetics like tissue uptake and clearance, which may exhibit non-linear behavior under different exposure conditions. To address these limitations, future refinements will focus on incorporating more anatomically and physiologically detailed compartmental structures within organs and integrating advanced mechanistic descriptions of nanoparticle interactions (e.g., active transport, protein corona effects). Based on our findings, specific future directions include expanding experimental data collection to encompass various exposure routes, dose levels, and more frequent time points. This is crucial for robust parameterization and validation, enabling the development of more comprehensive models applicable to diverse nanoparticle characteristics (e.g., size, shape, surface chemistry) and complex exposure scenarios. Such improvements will significantly enhance the model’s predictive ability and applicability in nanoparticle toxicokinetics. In the future, we plan to conduct further experiments to obtain additional data at more time points, which can be used to further improve the predictive ability of the model.

## 5. Conclusions

This study presents a novel PBTK modeling approach that specifically integrates a Hill equation-based model to describe the endocytosis of TiO_2_-NPs following inhalation exposure in rats, allowing for a mechanistic prediction of their biodistribution. The developed PBTK model demonstrated favorable agreement with published biokinetics in the lungs, liver, spleen, and kidneys, with correlation coefficients exceeding 0.85 (R^2^ > 0.85), validating the model’s robustness. Sensitivity analysis revealed that tissue–blood partition coefficients (P_lu_, P_li_, P_ki_) and the alveolar–lung transfer rate (K_alv_inter_) are key parameters significantly influencing TiO_2_-NPs distribution among organs. Despite the availability of two inhalation biodistribution datasets, the PBTK model developed here effectively predicts the distribution and accumulation of inhaled TiO_2_-NPs. With this model, it can predict the changes of TiO_2_-NPs content in rat body organs, including peak value and half-life. Finally, this modeling framework provides a predictive basis for evaluating TiO_2_-NPs behavior in vivo and holds significant potential for advancing hazard assessment of TiO_2_-NPs for human exposure, although it has some limitations, which should be improved in future.

## Figures and Tables

**Table 1:** Physiological parameters for the TiO_2_-NPs PBTK model [40].

Organ	Parameter	Description	Value	Units
Whole body	QC	Total blood flow	6.0345	L/h
	BW	Total body weight	0.263	kg
	TV	Total body volume	0.263	kg
Liver	V_li_	Liver volume	0.00983	L
	V_licab_	Liver capillary volume	0.00023	L
	V_litis_	Liver tissue volume	0.0096	L
	Q_li_	Liver blood flow	1.05	L/h
Lung	V_lu_	Lung volume	0.00167	L
	V_lucab_	Lung capillary volume	0.00047	L
	V_lutis_	Lung tissue volume	0.0012	L
	Q_lu_	Lung blood flow	6.0345	L/h
Kidney	V_ki_	Kidney volume	0.00221	L
	V_kicab_	Kidney capillary volume	0.00031	L
	V_kitis_	Kidney tissue volume	0.0019	L
	Q_ki_	Kidney blood flow	0.8509	L/h
Spleen	V_spl_	Spleen volume	0.00065	L
	V_splcab_	Spleen capillary volume	0.00012	L
	V_spltis_	Spleen tissue volume	0.00053	L
	Q_spl_	Spleen blood flow	0.0736	L/h
Rest of body	V_rob_	Rest of body volume	0.2613	L
	V_robcab_	Rest of body capillary volume	0.0102	L
	V_robtis_	Rest of body tissue volume	0.2511	L
	Q_rob_	Rest of body blood flow	4.06	L/h
Venous blood	V_ven_	Venous blood volume	0.0106	L
Arterial blood	V_art_	Arterial blood volume	0.0025	L

**Table 2:** Chemical-specific parameters for the TiO_2_-NPs PBTK model.

Organ	Parameter	Value	Units
Liver	X_li_	489.2806	dimensionless
	P_li_	47.4101	dimensionless
	K_limax_	263.8254	1/h
	K_li50_	0.3592	h
	K_liout_	19.4813	1/h
	K_lipcs_feces_	0.0884	1/h
Spleen	X_spl_	130.1283	dimensionless
	P_spl_	150	dimensionless
	K_splmax_	240	1/h
	K_spl50_	1000	h
	K_splout_	0.9507	1/h
Kidney	X_ki_	0.0199	dimensionless
	P_ki_	209	dimensionless
	K_kimax_	0.9929	1/h
	K_ki50_	13.6432	h
	K_kiout_	20	1/h
	K_kid_urine_	0.00005	1/h
Rob	X_rob_	0.0001	dimensionless
	P_rob_	10.8944	dimensionless
	K_robmax_	0.000001	1/h
	K_rob50_	0.008	h
	K_robout_	0.00005	1/h
Tracheobronchial	K_trach_feces_	0.3099	1/h
alveoli	K_alvpcs_trach_	8.3465	1/h
	K_alv_inter_	0.353	1/h
	K_inter_alv_	0.09	1/h
	K_alvout_	0.0109	1/h
	K_alv50_	202.1309	h
	K_alvmax_	0.000004	1/h
Lung	K_inter_max_	0.000050504	1/h
	K_inter50_	628.416	h
	K_interout_	0.0672	1/h
	P_lu_	30359	dimensionless
	X_lu_	49.1745	dimensionless
Upper airway	K_upp_robtis_	0.05	1/h
	K_upp_feces_	0.008	1/h

**Table 3:** Comparison of half-life between TiO_2_-NPs and Ti iron. The term “a” refers to the half-life value from the literature [46]. Half-lives were estimated by mono-exponential (log-linear) regression of the elimination phase (1–672 h).

Parameter	Liver (t_1/2_, h)	Kidney (t_1/2_, h)	Spleen (t_1/2_, h)
TiO_2_-NPs	452.8	179.6	350.7
Ti iron	1.9 ^a^	3.3 ^a^	2.1 ^a^

## Data Availability

Data will be made available on request.
